# IDDMSLD: An image dataset for detecting Malabar spinach leaf diseases

**DOI:** 10.1016/j.dib.2025.111293

**Published:** 2025-01-10

**Authors:** Adnan Rahman Sayeem, Jannatul Ferdous Omi, Mehedi Hasan, Mayen Uddin Mojumdar, Narayan Ranjan Chakraborty

**Affiliations:** Multidisciplinary Action Research Laboratory, Department of Computer Science and Engineering, Daffodil International University, Birulia, Dhaka 1216, Bangladesh

**Keywords:** Agriculture, Basella alba, Classification, Identification, Image processing, Plant pathology

## Abstract

Agriculture has always played a vital role in the economic development of Bangladesh. In Agriculture, leaf diseases have become an issue because they can lead to a major drop in both quality and quantity of crops. Therefore, leveraging technology to automatically detect diseases on leaves plays an important role in farming. Malabar Spinach (Basella alba) is a well-known, widely grown leafy vegetable, which is valued for its nutritional benefits. However, there is almost no dataset that can aid in identifying diseases affecting this important crop, which often leads to decreased quality as well as financial drawback. This lack of resources makes it difficult for farmers to recognize and manage common diseases. Our purpose is to solve this problem by creating a unique dataset of Bangladesh's Malabar Spinach leaves that will ease agricultural management and disease detection. Our dataset contains both healthy and diseased samples, categorised into four common ailments: Anthracnose, Bacterial Spot, Downy Mildew, and Pest Damage. We collected 3,006 original images in total. Images were collected from various locations in Bangladesh, including Mirpur, Savar, Sirajganj and Gazipur, with photographs taken under natural lighting conditions at different times of the day. This dataset will help the researchers for further research on Malabar Spinach disease detection implementing various efficient computational models and applying advanced machine learning techniques.

Specifications Table

**Table:** 

Subject	Computer Sciences, Agriculture Sciences.
Specific subject area	Computer Vision, Image Processing, Disease Classification, Machine Learning
Type of data	The images are in JPEG images (total 3006 images), with resolutions of 3000 × 4000-pixel dimensions and 96 × 96 dpi pixel density.
Data collection	This dataset comprises a total of 3006 Malabar Spinach leaf images, consisting both Healthy and diseased Leaf. Diseased leaves are categorised into 4 classes: Anthracnose, Bacterial Spot, Downy Mildew, and Pest Damage. The images were captured using: (i) Redmi Note 10 Lite, (ii) iPhone 15 Pro, and (iii)Redmi Note 9 smartphones.
Data source location	Data was collected from the following locations in Bangladesh: 1. Malabar Spinach field Mirpur-11, Dhaka-1206 (Latitude: 23°48′45.1″N, Longitude: 90°22′53.8″E) 2. Malabar Spinach field in Solonga, Sirajganj (Latitude: 24°25′08.7″N, Longitude: 89°30′23.5″E) 3. Malabar Spinach field in Khagan, Ashulia, Savar (Latitude: 23°52′32.1″N, Longitude: 90°19′42.0″E) 4. Malabar Spinach field Gazipur (Latitude: 24°04′14.8″N, Longitude: 90°32′32.3″E)
Data accessibility	Repository name: Mendeley Data Data identification number: 10.17632/sy69db2nz5.2 Direct URL to data: https://data.mendeley.com/datasets/sy69db2nz5/2

## 1 Value of the Data

• This dataset valuable because it provides a large, diverse collection of images of Malabar Spinach (Basella alba) affected by common diseases. This diverse collection will help in disease classification and modeling specifically for this crop as well as for the agricultural research and better disease detection techniques.
• Researchers can reuse this dataset to develop and refine machine learning models that can detect diseases early. These models could then be applied to other crops or expended with more data. Finally make it easier to implement automated disease detection system in agriculture on a large scale.
• This dataset contributes to progress in plant pathology by offering high-quality, labeled images. It allows researchers to test new ideas, develop better disease management strategies, and evaluate how well different AL model perform in identifying diseases.
• This dataset can be used for educational purposes. Students and researchers can use it to dive deeper into plant pathology, image processing, and AI Development. Making the dataset available means more opportunities for improving AL Models, ultimately benefiting farmers and the agricultural industry.

## 2 Background

Agriculture plays a vital role when it comes to a nation's growth. Likewise, Bangladesh's economic backbone is its agriculture [1]. Recent studies reveal that lesser-known and unexplored plants are excellent sources of nutrients and bioactive compounds suitable for both food and non-food use (Khan et al., 2011, 2015). Although its history is little known, experts believe the variety may have originated in Southeast Asia and India [2]. Malabar Spinach is widely cultivated in the rural areas of Bangladesh, but despite its value, several leaf diseases are harming the crop, negatively affecting both its yield and quality. It's a matter of regret that Bangladesh has not paid enough attention to modern technologies in cultivation [3]. Some of the common diseases are Anthracnose, Bacterial Spot, Downy Mildew, and Pest Damage, each of which poses a threat to plant health and productivity. Through early disease detection, we can achieve more efficient disease management and higher productivity. Malabar Spinach is a horticulture plant that can be grown in a small area space like a roof or garden [4]. This nutritious plant can be grown in small spaces with adequate light and soil moisture [5]. This dataset is valuable for both researchers and farmers. It addresses real challenges in detecting plant diseases. By focusing on Malabar Spinach, we fill the gap in agricultural datasets and lay the ground work for future advancement in managing crop diseases. This dataset is a valuable tool for studying plant health and developing methods for early disease detection.

## 3 Data Description

We classified our Malabar Spinach leaf images into a total of five classes. Each Class represents a specific conditions or type of leaf. Table 1 provides a detailed breakdown with the number of each class of the dataset.

**Table 1:** Statistics of the Malabar spinach dataset.

SL	Classes(Leaf)	Number of Images
**1**	Healthy	1399
**2**	Anthracnose	102
**3**	Bacterial Spot	752
**4**	Downy Mildew	240
**5**	Pest Damage	513

The dataset for Malabar Spinach disease detection comprises five classes of leaves: Healthy, Anthracnose, Bacterial Spot, Downy Mildew, and Pest Damage. We carefully labeled each class with high-quality images to ensure accurate classification. The dataset includes a total of 3006 images collected from diverse sources and covers a wide range of conditions to reflect real-world scenarios. Table 2 summarizes the dataset details, including the number of images per class, brief descriptions, and sample images to illustrate each category.

**Table 2:** Dataset summary of Malabar spinach.

Class	Description	Total images	Sample images
**Healthy Leaf**	Healthy leaves essential for photosynthesis and nutrient distribution, rich in vitamins, and prevent diseases, making them highly valuable for medical applications [6,7].	1399	
**Anthracnose**	Anthracnose is caused by Colletotrichum fungi in warm, wet climates. Early symptoms include water-soaked lesions [[8], [9], [10]].	102	
**Bacterial Spot**	Caused by *Pseudomonas* and *Xanthomonas* bacteria, spread through wind and rain in humid weather [11,12].	752	
**Downy Mildew**	Caused by *Sclerospora graminicola*. Symptoms include yellow patches and downy layers on leaves [13].	240	
**Pest Damage**	Identified by chewed areas and discoloration. Reduces photosynthetic efficiency and hinders growth [14].	513	

There are two dataset that are related to our, firstly, the dataset by Rahman et al. [15], titled, “Malabar Spinach dataset for disease classification using a deep learning approach,” consists of a total of 603 images, focusing on only two specific disease categories: Anthracnose Leaf Spot and Straw Mite. This dataset can be used for disease detection but it is limited to the mentioned two diseases. The “Local Spinach Leaf Dataset” by Hasan et al. [16], presents a set of images focused on leaf disease classification, consists of a total of 3999 images, classifying three types of spinach plants as follows: red spinach (fresh and non-fresh), water spinach (fresh and non-fresh), and Malabar spinach (fresh and non-fresh). The amount of data for each type of spinach plant is limited. The target of our dataset is to address this limitation by offering a comprehensive collection of Malabar Spinach leaf images, covering both healthy and diseased samples across four specific ailments. Our Dataset is much larger, containing 3006 images segmented into one healthy and four diseased classes, specifically designed to support the development of robust machine learning models for early disease detection. By improving disease management in Malabar Spinach, this dataset contributes to sustainable agriculture. This collection also gives researchers a starting point for creating sophisticated AI-powered diagnostic tools for tracking plant health. We have a total of 3006 original images in our Dataset, having both healthy and diseased leaves, where diseased leaves are categorized into 4 classes specifically designed to aid the development of robust machine learning models for early disease detection. Our dataset will be a revolutionary contribution to agriculture, as well as the researchers for creating sophisticated AI-driven diagnostic tools for tracking plant health.

We compared our dataset to evaluate the effectiveness and uniqueness with two closely related datasets from the works of Rahman et al. [15] and Hasan et al. [16]. Table 3 provides a detailed comparison of the datasets. We highlighted the numbers of images specific to classes. Our dataset includes a total of 3006 images dividing into 5 classes categorized as Healthy, Anthracnose, Bacterial Spot, Downy Mildew and Pest Damage, showing both Healthy and Diseased images. The other datasets have fewer categories. The closest datasets that can be compared with ours are given in Table 3.

**Table 3:** Comparison with available datasets of Malabar spinach.

SL	Classes	Number of images		
		Our Dataset	Rahman et al. [15]	Hasan et al. [16]
**1**	Healthy-Leaf	✔ (1399)	✔ (not mentioned)	✔(not mentioned)
**2**	Anthracnose	✔ (102)	✔ (not mentioned)	X
**3**	Bacterial Spot	✔ (752)	X	X
**4**	Downy Mildew	✔ (240)	X	X
**5**	Pest Damage	✔ (513)	✔ (not mentioned)	X

[Note: ✔ = Data available in Mendeley Repository].

We collected our data throughout October and November 2024. We captured leaf images in different days and in different time of the day. Location was different so that we can cover various types. We recorded key parameters including the class of leaves, weather conditions, date, time, temperature, humidity and the devices used for image capture. Details are given in Table 4.

**Table 4:** Collection details of Malabar spinach dataset.

Malabar Spinach Class Name	Weather	Date	Time	Temperature (°C)	Humidity (%)	Camera Device	Location
Bacterial Spot Leaf	Sunny	17 October 2024	Morning	27 °C	89 %	Redmi Note 10 Lite (100 %)	Solonga, Sirajgonj
Anthracnose Leaf	Windy	17 October 2024	Afternoon	26 °C	82 %	Redmi Note 10 Lite (65 %) and iPhone 15 Pro (35 %)	Solonga, Sirajgonj
Healthy Leaf	Sunny	18 October 2024	Morning	31 °C	92 %	iPhone 15 Pro (50 %) and Redmi Note 9 (50 %)	Khagan, Ashulia, Savar
Pest Damage Leaf	Sunny	18 October 2024	Noon	30 °C	82 %	Redmi Note 10 Lite(45 %) iPhone 15 Pro (25 %) and Redmi Note 9 (40 %)	Khagan, Ashulia, Savar
Anthracnose Leaf	Cloudy	21 October 2024	Morning	29 °C	88 %	Redmi Note 10 Lite (50 %) and iPhone 15 Pro (50 %)	Khagan, Ashulia, Savar
Downy Mildew Leaf	Cloudy	01 November 2024	Afternoon	27 °C	70 %	Redmi Note 10 Lite (60 %)	Khagan, Ashulia, Dhaka
Bacterial Spot Leaf	Cloudy	02 November 2024	Morning	29 °C	78 %	Redmi Note 10 Lite (100 %)	Mirpur-11, Dhaka
Downy Mildew Leaf	Cloudy	02 November 2024	Afternoon	28 °C	69 %	Redmi Note 10 Lite (50 %) and Redmi Note 9 (50 %)	Mirpur-11, Dhaka

Due to several practical limitations, we could not implement real-time data collection and the use of specialized devices for automated monitoring was not achievable for our study. Significant technical resources are needed to develop real-time system or building dedicated devices, which were outside of the scope of the study. Additionally, it is challenging to set up monitoring devises in the rural and remote areas. That's why we chose to collect data manually. This gave us better control over the quality and variety of samples. This approach gave us the flexibility on capturing image and organize them

## 4 Experimental Design, Materials and Methods

### 4.1 Methodology

**Fig. 1:**
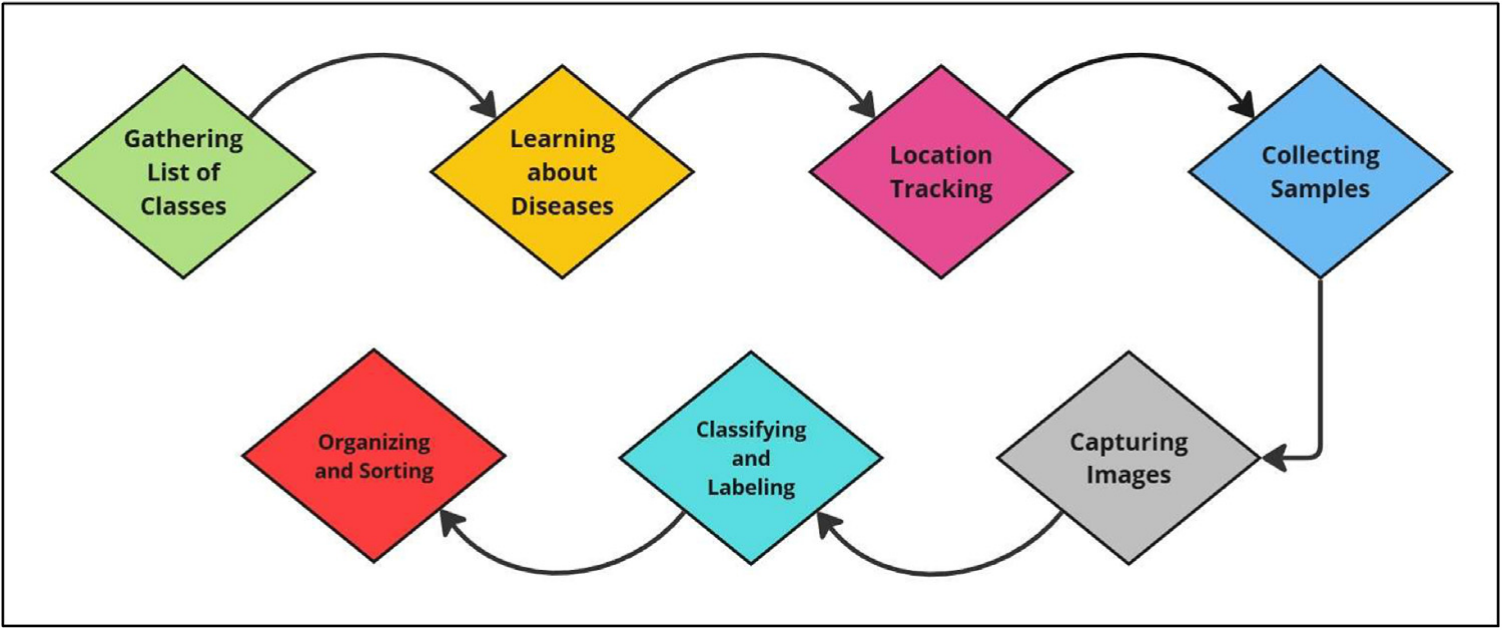
Process of steps of the whole workflow.

In Fig. 1, we presented our methodology outlining the systematic steps taken to develop a robust and unique dataset, ensuring the accuracy of our study. We began by identifying key diseases and gathering insights from experts, followed by recording field locations and temperatures, and collecting a variety of leaf samples. Detailed, high-quality images were captured and labelled according to their respective classes. Finally, the dataset was augmented and organized folder-wise to facilitate analysis and model training.

1. *Gathering List of Classes:* After selecting our goal, our first task was to list the common diseases that harm the Malabar Spinach plant the most. We conducted research on the internet and consulted with local experts to gain a deeper understanding of these diseases.
2. *Learning About Diseases:* To understand symptoms, reasons and effects of each disease we had to learn about Malabar Spinach plants. An agricultural specialist and plant pathologist guided us on field to gain proper knowledge about each disease's identification and classification. We also gained knowledge to collect effective data.
3. *Location Tracking:* We needed to keep track of each Malabar field's location for future reference and model improvements. We used GPS system to log them. Noting these locations helped us ho environmental and farming differences affect the plants.
4. *Collecting Samples:* We gathered a large variety of Malabar spinach leaves from different growth stages to cover a range of appearance. We captured both heathy and diseased leaves from various fields to ensure that our samples were diverse.
5. *Capturing Images:* We used high-resolution smartphones to take detailed photos of each leaf. To gather detailed images, we placed each leaf on a white paper for its background.
6. *Classifying and Labeling:* After collecting all images, we sorted the images into different disease categories and labeled them accordingly. To ensure we have categorized perfectly one of our teammates re-checked the images. We had to delete many images because of blurriness.
7. *Organizing and Storing Data:* At first, we stored all our data on our personal smart phones. To process them all together we gathered them in single computer later on.

### 4.2 Image preprocessing and classification

**Fig. 2:**
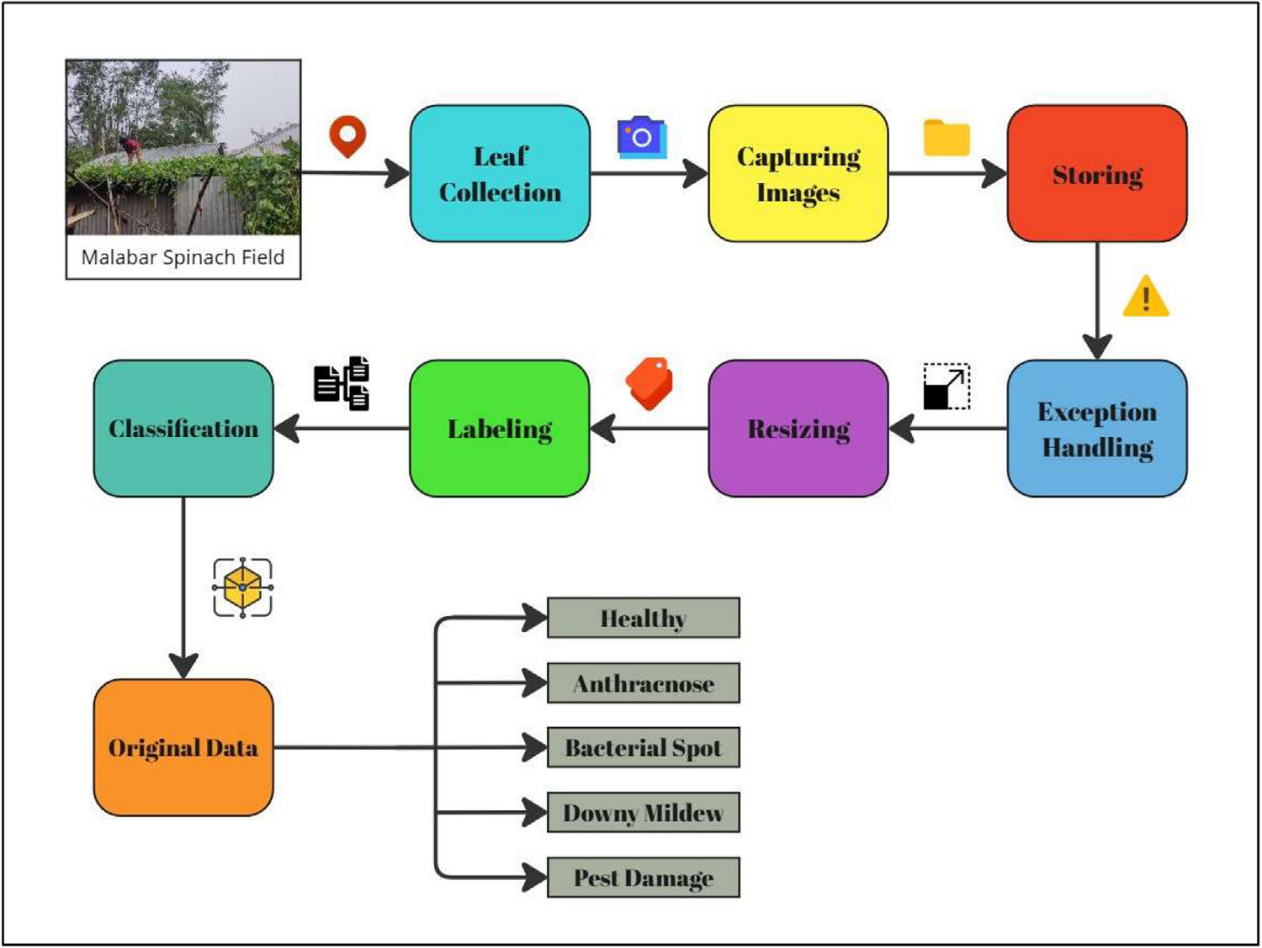
Workflow of image preprocessing and classification.

In Fig. 2, we followed a structured image preprocessing workflow to prepare our dataset. This workflow involved systematic steps starting from field visits for leaf collection and image capturing to storage, resizing, labeling and classification. Each step was carefully executed to ensure the dataset's quality and diversity, enabling its effective use in future research and analysis.

1. *Malabar Spinach Field:* We went to our listed locations and identified the Malabar Spinach plant. We carefully selected the leaves to ensure we got a variety of healthy and diseased leaves for our study, representing different conditions for better analysis.
2. *Leaf Collection:* We collected leaves from plants manually, ensuring they were affected by diseases and healthy ones. We carefully handled the leaves, avoiding any physical damage.
3. *Capturing Images:* We used devices with high-resolution cameras to capture the leaf images. All images were taken in natural light, avoiding shadows and reflections to maintain quality for accurate classification.
4. *Storing Images:* After collecting all images, we merged them into a single device (Computer). A single storage location helped us control every image individually and collectively.
5. *Exception Handling:* We reviewed each image after storing them on the computer. We faced some errors; for instance, very few images had shadows, and some were blurry. We had to remove the background and make it white manually for some images.
6. *Resizing:* Since we captured images from multiple sources, the image ratios weren't the same. We resized images to a particular ratio, for instance, 3000 × 4000 pixels.
7. *Labeling:* We assigned labels to each image based on their condition, such as Healthy, Anthracnose, or Pest Damage. Accurate labeling will help us with further model training and classifying models.
8. *Classification:* After labeling each image, we classified every image into separate folders based on their classes. This step simplifies data management and prepares the dataset for augmentation.

We followed this systematic workflow to ensure that the dataset is carefully curated and organized. We maintained high quality and consistency throughout the process by addressing key steps such as handling, resizing, and labeling. After completing all the processes, we collected a total of 3006 Malabar Spinach images, including both healthy and unhealthy leaves. This structured approach enhances the dataset's usability and ensures its relevance for future research in agricultural studies.

## Limitations

From a geological perspective, our dataset is limited to specific areas of Bangladesh. In the dataset, some categories have fewer samples due to limited availability, although the data can be expanded using augmentation methods. Additionally, we classified our data through visual verification, so there may be some minor errors. The dataset focuses on static images rather than real-time data, which could offer additional insights but it proved impractical due to logistical challenges and resource limitations.

## Ethics Statement

We ensure that our study was conducted in full compliance with ethical guideline. No harm was caused to plants, animals, or humans. No data was sourced from social media platforms. All authors confirm adherence to ethical standards required for publication in Data in Brief.

## CRediT Author Statement

**Adnan Rahman Sayeem:** Conceptualization, Software, Data curation. **Jannatul Ferdous Omi:** Writing, Visualization. **Mehedi Hasan:** Data curation, Methodology. **Mayen Uddin Mojumdar:** Supervision, Methodology, Review. **Narayan Ranjan Chakraborty:** Visualization, Investigation.

## Data Availability

Mendeley DataMalabar Spinach Disease Detection Dataset (Original data).
